# Language Functional Connectivity Alterations During Resting State in Brain Arteriovenous Malformation Patients

**DOI:** 10.1111/cns.70602

**Published:** 2025-09-05

**Authors:** Xiaofeng Deng, Qiji Shi, Zekun Han, Shuo Wang, Yong Cao, Xiaolin Chen, Yan Zhang, Bo Wang, Minyu Jian, Fangrong Zong, Jizong Zhao

**Affiliations:** ^1^ Department of Neurosurgery Beijing Tiantan Hospital, Capital Medical University Beijing China; ^2^ China National Clinical Research Center for Neurological Diseases Beijing China; ^3^ School of Artificial Intelligence Beijing University of Posts and Telecommunications Beijing China; ^4^ State Key Laboratory of Brain and Cognitive Science, Beijing MRI Center for Brain Research, Institute of Biophysics Chinese Academy of Sciences Beijing China; ^5^ University of Chinese Academy of Sciences Beijing China; ^6^ Department of Anesthesiology Beijing Tiantan Hospital, Capital Medical University Beijing China

**Keywords:** arteriovenous malformation, functional connectivity, language, resting state, track‐weighted static functional connectivity

## Abstract

**Objectives:**

Unruptured brain arteriovenous malformations (AVMs) typically do not cause aphasia, even when the traditional language areas are affected by the nidus. We attempted to elucidate its language reorganization mechanism by analyzing the alterations in functional connectivity using functional connectivity (FC) and track‐weighted static functional connectivity (TW‐sFC) approaches.

**Methods:**

This cross‐sectional study prospectively enrolled patients with AVMs involving left‐hemisphere language areas and healthy controls. All participants underwent resting‐state functional magnetic resonance imaging (rs‐fMRI) and diffusion tensor imaging scans. Conventional FC analysis was used to investigate the spatially segregated functional connectivity in the gray matter, and the TW‐sFC method was applied to explore the functional connectivity constrained by the white matter.

**Results:**

34 AVM patients with lesions involving the left cerebral hemisphere and 27 healthy subjects were included. FC analysis findings revealed decreased FC intensity between the left‐hemisphere language‐associated regions and their right‐hemisphere homologs in AVM patients. Additionally, increased FC intensity was observed between the anterior cingulate cortex (ACC) and the language‐related areas in bilateral cerebellar hemispheres (lobule VIII, VIIb, Crus I, and Crus II). The TW‐sFC results demonstrated increased intensity in multiple right‐hemisphere fiber bundles, the left anterior thalamic radiation (ATR) and the callosum.

**Conclusions:**

Three factors may contribute to maintaining intact language function in AVM patients, including the weakened inhibitory effect from the left dominant cerebral hemisphere over the right cerebral hemisphere leading to activation of the potential language functions of the right cerebral hemisphere (inter‐cerebral connection reorganization), the functional upregulation of the cerebral language areas by cerebellar language‐related brain regions via ACC (cerebrocerebellar connection reorganization), as well as the enhanced functions of the brain areas surrounding the lesion in the left cerebral hemisphere (intracerebral connection reorganization).

**Trial Registration:**

This study is registered in the Chinese Trial Registry (clinical trial number: ChiCTR1900020993)

## Graphical abstract

Arteriovenous malformations involving the language area usually do not lead to language dysfunction, so it provides a unique model for studying language reorganization. Three factors may contribute to maintaining intact language function in AVM patients: inter‐cerebral connection reorganization; cerebrocerebellar connection reorganization; intracerebral connection reorganization.

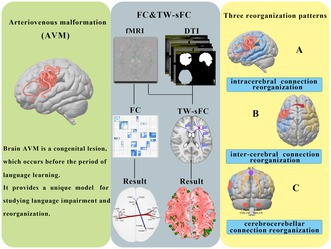

**Abbreviations**
AAL: automated anatomy labeling
ACC: anterior cingulate cortex
ACT: anatomically constrained tractography
AF: arcuate fasciculus
AFQ: automatic fiber quantification
AG: angular gyrus
AQ: aphasia quotients
ATR: anterior thalamic radiation
AVM: arteriovenous malformation
BOLD: blood oxygen level‐dependent
CO: central opercular cortex
CSD: constrained spherical deconvolution
DTI: diffusion tensor imaging
EPI: echo‐planar imaging
FC: functional connectivity
FDR: false discovery rate
fMRI: functional magnetic resonance imaging
FMRIB: functional magnetic resonance imaging of the brain
FO: frontal operculum
FOD: fiber orientation distribution
FSL: FMRIB software library
FWE: family‐wise error
FWHM: full‐width at half‐maximum
HG: Heschl's Gyrus
IFG: inferior frontal gyrus
IFOF: inferior fronto‐occipital fasciculus
ILF: inferior longitudinal fasciculus
ITG: inferior temporal gyrus
MNI: Montreal Neurological Institute
MP‐RAGE: magnetization‐prepared rapid‐acquisition gradient‐echo
MTG: middle temporal gyrus
Pa CiG: paracingulate gyrus
PO: parietal operculum cortex
PP: planum polare
PT: planum temporale
ROI: region of interest
rs‐fMRI: resting state functional magnetic resonance imaging
SIFT: spherical‐deconvolution informed filtering of tracks
SLF: superior longitudinal fasciculus
SLFT: superior longitudinal fasciculus temporal part
SNR: signal‐to‐noise ratio
STG: superior temporal gyrus
TW‐sFC: track‐weighted dynamic static connectivity
UF: uncinate fasciculus
WAB: Western Aphasia Battery

## 1 Introduction

Language network alterations are widely present in patients with intracranial lesions, whether they have or do not have language dysfunctions [1, 2, 3]. The observed changes in the language network may be the result of language function damage caused by the lesion, the reason for patients to maintain normal language function (functional remodeling), or both. Given that language learning and the establishment of language networks occur primarily postnatally, overall, lesions affecting language areas fall into two etiological categories: acquired pathologies (e.g., gliomas, strokes, traumatic brain injuries) typically emerging after language network maturation [3, 4], and congenital conditions (e.g., brain arteriovenous malformations [AVMs], perinatal infarcts) developing before the period of language network establishment [5, 6]. Previous studies have identified notable variations in the patterns and degrees of language reorganization between congenital and acquired diseases [7, 8, 9]. For example, a common and interesting clinical phenomenon is that if the lesion involves the language cortex, most glioma patients experience language dysfunction, while unruptured AVM patients rarely show language impairment [10, 11]. Compared to acquired lesions, congenital intracerebral lesions are rare and the language reorganization mechanism needs further exploration [5, 12]. We believe congenital lesions provide a unique model and a new perspective for studying language network reconfiguration.

Previously, we studied the functional alterations and language‐related white matter changes of AVM patients using task‐based functional magnetic resonance imaging (fMRI), resting state fMRI (rs‐fMRI), and diffusion tensor imaging (DTI) [7, 8, 12, 13, 14, 15]. Part of the language remodeling mechanism has been elucidated. For example, analysis of low frequency fluctuation amplitude (ALFF) revealed increased values in the right cerebral and left cerebellar hemispheres, indicating the related cortex in the nondominant hemispheres was recruited in the reorganized language network [12]. Complementary DTI analyses demonstrated lesion‐induced impairment of language‐associated fascicles in the left hemisphere alongside remodeling of other language‐related fascicles in both cerebral hemispheres detected by the automatic fiber quantification (AFQ) study [15]. Based on the results of our previous studies, we found three possible mechanisms of language reorganization, including the remodeling of the brain areas surrounding the lesion in the left cerebral hemisphere, the homologous areas of the traditional left language areas in the right cerebral hemisphere, and the cerebellar hemispheres. Moreover, language reorganization exists in both gray and white matter. However, further studies are needed to confirm this hypothesis and to detail the language impairment and remodeling mechanisms.

Functional connectivity (FC), which measures temporal correlations in neural activity between gray matter regions [16], has been extensively studied in language dysfunction caused by brain disorders. For instance, an FC study of language function changes in children with epilepsy revealed increased right frontal FC integration and greater language processing load on the right middle frontal gyrus, indicating compensatory language plasticity [17]. Given that specific types of language impairments correlate with aberrant activity patterns in brain networks, FC can identify distinct linguistic deficit profiles in post‐stroke aphasia [18]. While resting‐state FC alterations provide unique perspectives on language networks, their investigation in AVM contexts remains notably limited, necessitating further investigation of FC alterations in these settings. Concurrently, track‐weighted static functional connectivity (TW‐sFC) has emerged as a method integrating structural and functional connectivity data [19]. By projecting functional connectivity onto white matter pathways, TW‐sFC quantifies static correlations between gray matter regions anatomically connected via specific white matter tracts [20]. Critically, the application of TW‐sFC to AVM research has not yet been explored. Aside from the ALFF technique for fMRI analysis and AFQ techniques for DTI analysis we used in previous studies, we believe the research on language functional connectivity alterations, as well as the coupling of structure and function information in AVM patients will complement and reinforce our hypothesis.

Both FC and TW‐sFC methods were applied in the current study. The study of FC analysis is crucial for understanding the organization and function of the brain, as it provides insight into the large‐scale neural networks that underlie cognition and behavior [21]. However, traditional FC shows the arbitrary distribution of connectivity throughout the brain, lacking the anatomical support to determine the validity of these connections within our brain's structural framework [20]. An alternative approach, TW‐sFC, integrates structural and functional information, revealing changes along the white matter pathways, i.e., sub‐cortical regions. Nevertheless, the result of TW‐sFC represents the joint effect of functional signals in the gray matter areas and may not directly reflect specific functional regions [19]. Therefore, FC and TW‐sFC complement each other in this study. FC offers a broader perspective on brain connectivity across the entire brain, revealing co‐activation regions under resting state. Meanwhile, TW‐sFC enhances the analysis by incorporating the constraints of structural information derived from the DTI data, thus refining the understanding of multiple functional connections.

In this study, we processed resting‐state fMRI (rs‐fMRI) data using functional connectivity (FC) analysis to examine functional connectivity alterations in AVM patients. Meanwhile, we applied the TW‐sFC approach to integrate rs‐fMRI and diffusion MRI (dMRI) data, investigating the coupling between functional connectivity and language‐related white matter pathways. By combining these multimodal approaches, our study characterizes language network alterations in AVM patients across cortical and subcortical levels. This integrated framework aims to elucidate the mechanisms of language reorganization in AVM patients and explain the paradoxical phenomenon of preserved language function despite lesion encroachment on canonical language areas.

## 2 Materials and Methods

### 2.1 Subjects

This study was approved by the Institutional Review Board of Beijing Tiantan hospital, Capital Medical University (number: KY2018‐103‐01), and registered in the Chinese Trial Registry (clinical trial number: ChiCTR1900020993). Written informed consent was obtained from each participant. Prior to this study, we have analyzed information from the database, and several papers regarding the task‐based fMRI, ALFF, and DTI have been published [12, 13, 15].

All patients were enrolled at Beijing Tiantan Hospital. AVM lesions were anatomically localized to the left hemisphere, specifically involving the frontal lobe (inferior and middle frontal gyrus, and precentral gyrus), the temporal lobe (superior, middle and inferior temporal gyrus, and temporal pole) or the parietal lobe (supramarginal gyrus and angular gyrus). Other detailed inclusion and exclusion criteria were described in our previous paper regarding white matter plasticity in AVM patients [15].

Moreover, age‐ and sex‐matched healthy subjects (the control group) were recruited as controls.

### 2.2 Language Function Evaluation

We administered the Chinese version of the Western Aphasia Battery (WAB) to assess language functions in all participants. This comprehensive aphasia assessment tool has been standardized on large representative samples. Although its ability to differentiate specific subtypes (e.g., anomic aphasia and primary progressive aphasia) is limited, the Chinese version is widely adopted in clinical practice [22, 23]. This acceptance primarily stems from two key advantages: its approximately one‐hour administration time maintains clinical feasibility for most aphasia patients while covering major aphasia classifications [22, 23]. Scores from its four core domains, including fluency, comprehension, repetition, and naming, were separately compared between the AVM group and the control group. Moreover, the aphasia quotient (AQ) score (range 0–100) was calculated to quantify global language function [13].

### 2.3 Image Acquisition

MRI data of all participants were acquired using a 3‐T Prisma‐fit scanner (Siemens, Erlangen, Germany) with a 20‐element head–neck coil at the Institute of Biophysics, Chinese Academy of Sciences. The MRI scans included the following contents, and the detailed scanning parameters can be found in our previously published articles [12, 15].

1. Three‐dimensional T1‐weighted images, which were acquired with a magnetization‐prepared rapid‐acquisition gradient‐echo (MP‐RAGE) sequence.
2. The rs‐fMRI data, acquired with an interleaved T2‐weighted axial gradient‐echo echo‐planar imaging (EPI) sequence.
3. DTI datasets, acquired using a fully sampled spin‐echo EPI sequence.

### 2.4 Data Processing

**FIGURE 1:**
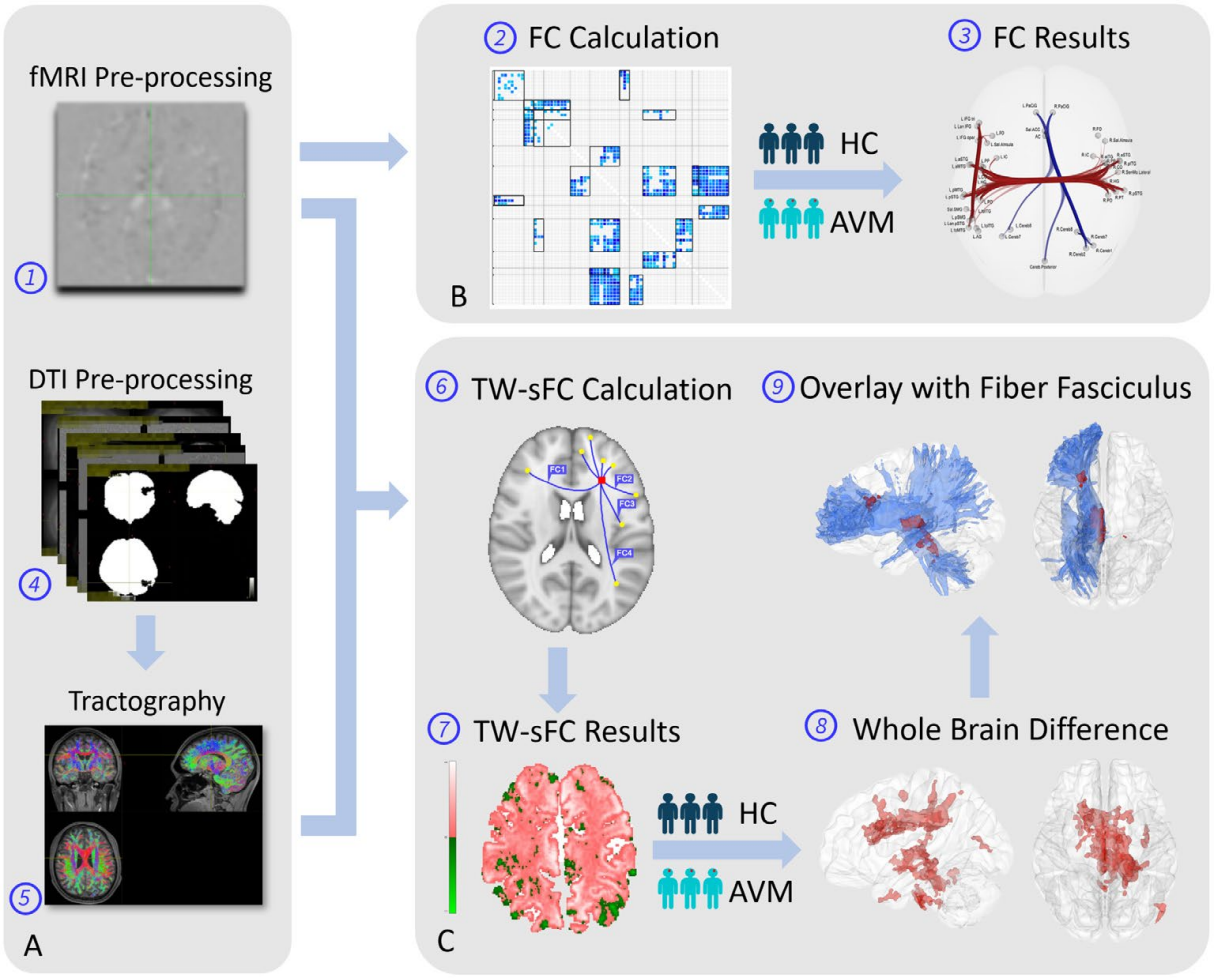
Analysis pipeline. Initially, fMRI data was preprocessed (1) to conduct the functional connectivity analysis (2) and compared between two groups to detect the whole brain connectivity difference (3). Subsequently, DTI raw data were preprocessed (4) to generate whole‐brain tractography (5). The whole‐brain fiber tractography and preprocessed fMRI data were integrated using TW‐sFC techniques (6) to produce a whole‐brain TW‐sFC map for each subject (7). These TW‐sFC results were then compared between groups to calculate the whole brain voxel‐wise differences (8). Finally, the whole brain difference was then overlapped with the fiber template to investigate the relationship between the spatial distribution of TW‐sFC results and locations of white matter fasciculus. Panel A, data preprocessing and tractography. Panel B, FC analysis. Panel C, TW‐sFC analysis.

We explored the reorganization mechanisms among AVM patients through two publicly available methodologies. The FC technique was applied to identify the network of gray matter activity connections using fMRI data [21]. Moreover, the TW‐sFC approach integrating the fMRI and DTI data was used to enhance the structural foundation of FC. The data processing pipeline is illustrated in Figure 1.

#### 2.4.1 Lesion Mapping

The brain region impaired by the AVM lesions was manually delineated slice‐wise for each patient using T1‐weighted MP‐RAGE images via MRIcron [12, 15]. This process was conducted by one neurosurgeon (Dr. Xiaofeng Deng) and verified by two senior neurosurgeons (Drs. Xiaolin Chen and Yan Zhang). Subsequently, the location of each lesion was standardized to the Montreal Neurological Institute (MNI) space utilizing the Functional Magnetic Resonance Imaging of the Brain (FMRIB) Software Library (FSL) (https://fsl.fmrib.ox.ac.uk/fsl/fslwiki/). AVM patients were then classified into the frontal, temporal, and parietal subgroups based on the lesion's location. Finally, separate images were generated for the AVM group and its three subgroups, accounting for any overlapping lesion locations.

#### 2.4.2 Data Preprocessing

The software used for data preprocessing includes MRtrix3 version 3.0.3 (64‐bit release) and Functional Magnetic Resonance Imaging of the Brain (FMRIB) Software Library (FSL) toolbox version 6.0.5.2 [24, 25]. The preprocessing of T1‐weighted images included skull stripping followed by segmentation into a 5‐tissue‐type (5TT) image, preparing for subsequent tractography analysis. Additionally, T1‐weighted images were registered to the 1‐mm resolution MNI 152 templates using the FSL toolbox.

We first realigned the DTI data to T1‐weighted images and then preprocessed DTI data using MRtrix3, which involved denoising, Gibbs' artifacts unringing, motion and distortion correction, and brain mask estimation [20, 21].

The fMRI data was also registered to T1‐weighted images in MNI space. Afterwards, pre‐processing steps including removal of the first four out of 144 time points in the time series and slice timing correction were applied. Motion correction was performed using rigid‐body transformations. Temporal filtering was implemented to attenuate high‐frequency noise in fMRI data via Gaussian smoothing, with the filtering threshold set between 0.01 Hz and 0.1 Hz. To enhance the signal‐to‐noise ratio (SNR), a 6 mm full‐width at half‐maximum (FWHM) spatial smoothing was subsequently applied [21].

#### 2.4.3 FC Analysis

FC analysis refers to the temporal correlation of blood oxygen level‐dependent (BOLD) signals between distinct brain regions [26, 27]. After preprocessing the fMRI data, we computed functional connectivity using the CONN toolbox pipeline (https://web.conn‐toolbox.org/). Specifically, we employed a subset consisting of 132 regions and 32 network‐level regions of interest (ROIs) from both the Harvard‐Oxford Atlas (maximum likelihood cortical and subcortical atlases) and the Automated Anatomy Labeling (AAL) Atlas for connectivity calculation [28, 29].

#### 2.4.4 Tractography and TW‐sFC Analysis

Following the preprocessing of dMRI data, we performed probabilistic tractography via the MRtrix3 software. Single‐shell 3‐tissue constrained spherical deconvolution (CSD), Anatomically Constrained Tractography (ACT) and spherical‐deconvolution informed filtering of tracks (SIFT) were applied to generate 1 million streamlines for each subject algorithm [30, 31, 32].

The whole brain tractography was then combined with the pre‐processed fMRI data to calculate the whole brain TW‐sFC intensities. In specific, each voxel's TW‐sFC intensity is obtained by averaging the FC across all tracts traversing that voxel [26]. Each tract FC values were assigned by the functional signal correlation of two endpoints. Once the whole brain TW‐sFC map was generated for each participant, it was registered to the MNI template using a non‐linear algorithm. Spatial differences between the AVM group and the healthy group were computed using an unpaired two‐sample *t*‐test. The name of the differential brain region, the size of the cluster, and the MNI spatial coordinates of the whole‐brain peak values were listed in Table S1. These differences were then superimposed onto the fiber template in the Johns Hopkins University (JHU) white‐matter tractography atlas. To detect significant variations in fiber bundles, we quantified the voxel‐wise overlap on the fiber template. However, when applying cluster‐level thresholding to identify meaningful results, an overly lenient threshold produced fragmented clusters with anatomically disorganized distributions, whereas an excessively stringent threshold yielded insufficient positive findings, risking false negatives. Therefore, clusters exceeding 200 voxels were deemed significant and reported.

The JHU white‐matter tractography atlas comprises 11 pairs of fasciculi for both hemispheres, including the anterior thalamic radiation (ATR), the inferior fronto‐occipital fasciculus (IFOF), the inferior longitudinal fasciculus (ILF), the uncinate fasciculus (UF), the superior longitudinal fasciculus (SLF), the superior longitudinal fasciculus temporal part (SLFT), the cingulum hippocampus (CH), the cingulum cingulate gyrus (CC), the corticospinal tract (CST), the forceps minor and the forceps major. Among these, the ATR, IFOF, ILF, UF, SLF and SLFT were supposed to be associated with language function [15]. Additionally, a callosum region of interest from the Johns Hopkins University ICBM‐DTI‐81 White‐Matter Atlas was incorporated into our study to reflect the interhemispheric connection [27].

#### 2.4.5 Statistical Analysis

To identify significant differences of FC between AVM patients and healthy controls, we applied cluster‐level false discovery rate (FDR) correction with a threshold of *p* < 0.05. For TW‐sFC analysis, voxel‐wise spatial comparisons were performed using SPM12 (https://www.fil.ion.ucl.ac.uk/spm/software/spm12/) to detect spatial differences between the two groups. Cluster‐level family‐wise error (FWE) correction with a significance threshold of *p* < 0.005 was applied in this analysis. Categorical variables (e.g., gender) were analyzed using the chi‐square test. For continuous data such as age and WAB scores, normality was assessed via the Shapiro–Wilk test. Normally distributed data were compared via independent samples *t*‐test for group comparisons, while non‐normally distributed data were analyzed using the non‐parametric Mann–Whitney *U* test.

## 3 Results

### 3.1 Demographic Characteristics

We finally enrolled 34 AVM patients (the AVM group) with lesions involving left‐hemisphere language areas. There were 18 males and 16 females with ages ranging from 18 to 51 years (Mdn = 29.5; IQR = 24.5–37). The most common clinical manifestations were headache (*n* = 12), seizures (*n* = 6), dizziness (*n* = 5), transient numbness of limbs (*n* = 3), and asymptomatic presentation (*n* = 8). According to the WAB test, no patient presented with aphasia (Table 1). Meanwhile, 27 healthy subjects (the control group) were recruited as controls. There was no significant difference in sex, age, or the four domains of WAB results between the AVM group and the control group.

**TABLE 1:** Demographic data and WAB results of different groups.

Group	Sex (male:female)	Age (years)	WAB results				
			Spontaneous speech (range 0–20)	Auditory comprehension (range 0–200)	Repetition (range 0–100)	Naming (range 0–100)	Aphasia Quotient (AQ) (range 0–100)
**Control group**	13:14	27.22 ± 7.22	19.94 ± 0.14	197.93 ± 3.21	99.56 ± 1.01	98.81 ± 1.14	99.35 ± 0.49
**AVM group**	18:16	30.97 ± 9.13	19.88 ± 0.30	197.35 ± 3.71	99.58 ± 1.08	98.76 ± 1.10	99.16 ± 0.70
*Z*/*χ* ^2^ values^a^	0.138	−1.840	−0.620	−0.648	−0.564	−0.208	−0.997
*p* ^a^	0.710	0.066	0.535	0.517	0.573	0.836	0.319
**Frontal AVM subgroup**	7:7	31.07 ± 7.01	19.89 ± 0.19	196.71 ± 3.29	99.43 ± 1.22	98.71 ± 1.27	99.107 ± 0.53
*Z*/*χ* ^2^ values^a^	0.013	−1.944	−1.041	−1.257	−0.259	−0.202	−1.667
*p* ^a^	0.910	0.052	0.488	0.269	0.860	0.860	0.108
**Temporal AVM subgroup**	6:4	29.90 ± 8.09	19.96 ± 0.13	197.20 ± 5.01	99.60 ± 1.26	99.00 ± 1.05	99.36 ± 0.57
*Z*/*χ* ^2^ values^a^	0.410	−0.978	−0.375	−0.242	−0.507	−0.390	−0.123
*p* ^a^	0.787	0.335	0.827	0.853	0.749	0.749	0.906
**Parietal AVM subgroup**	5:5	31.90 ± 12.81	19.80 ± 0.51	198.40 ± 2.80	99.80 ± 0.63	98.60 ± 0.97	99.08 ± 1.01
*Z*/*χ* ^2^ values^a^	0.010	−0.944	−0.481	−0.310	−1.154	−0.631	−0.507
*p* ^a^	0.920	0.353	0.775	0.801	0.048	0.602	0.699

^a^ Compared with the control group, the analysis of gender differences employed the chi‐square test. For other datasets that did not conform to a normal distribution, non‐parametric tests were utilized.

**FIGURE 2:**
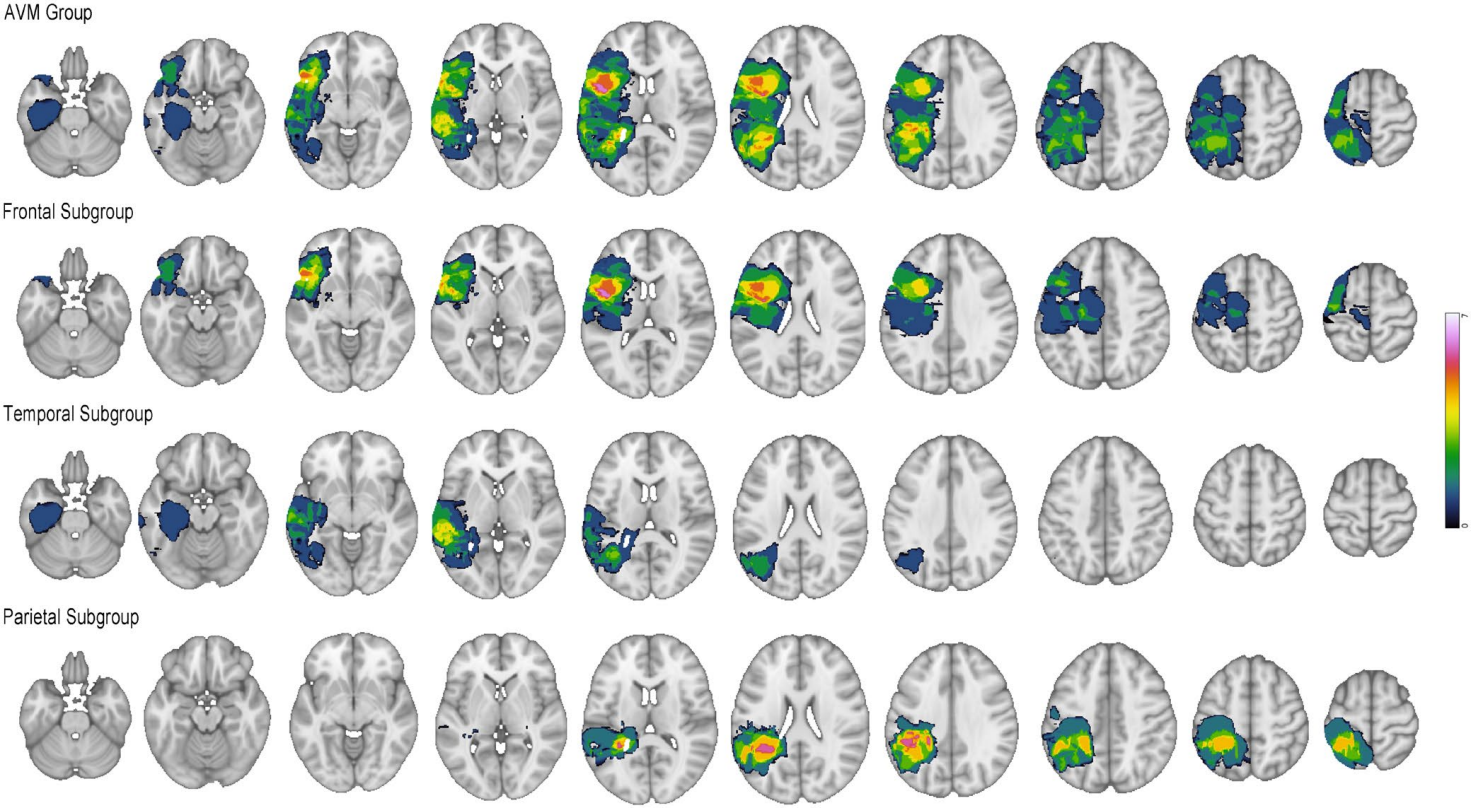
AVM Lesion heatmap. Each row illustrates several slices of lesion maps corresponding to different groups. The first row depicts the AVM group, followed by the frontal subgroup in the second row, the temporal subgroup in the third row, and finally, the parietal subgroup in the fourth row. The color bar corresponds to the number of lesions observed within this specific area across patients.

In the AVM group, there were 14 patients with lesions located in the left frontal lobe (the frontal subgroup), 10 in the left temporal lobe (the temporal subgroup), and 10 in the parietal lobe (the parietal subgroup) (Figure 2). Similarly, compared to the control group, no significant difference was observed in sex, age, or the four domains of WAB results in the three subgroups (Table 1).

### 3.2 Results of FC Analysis

#### 3.2.1 Results of the AVM Group

**FIGURE 3:**
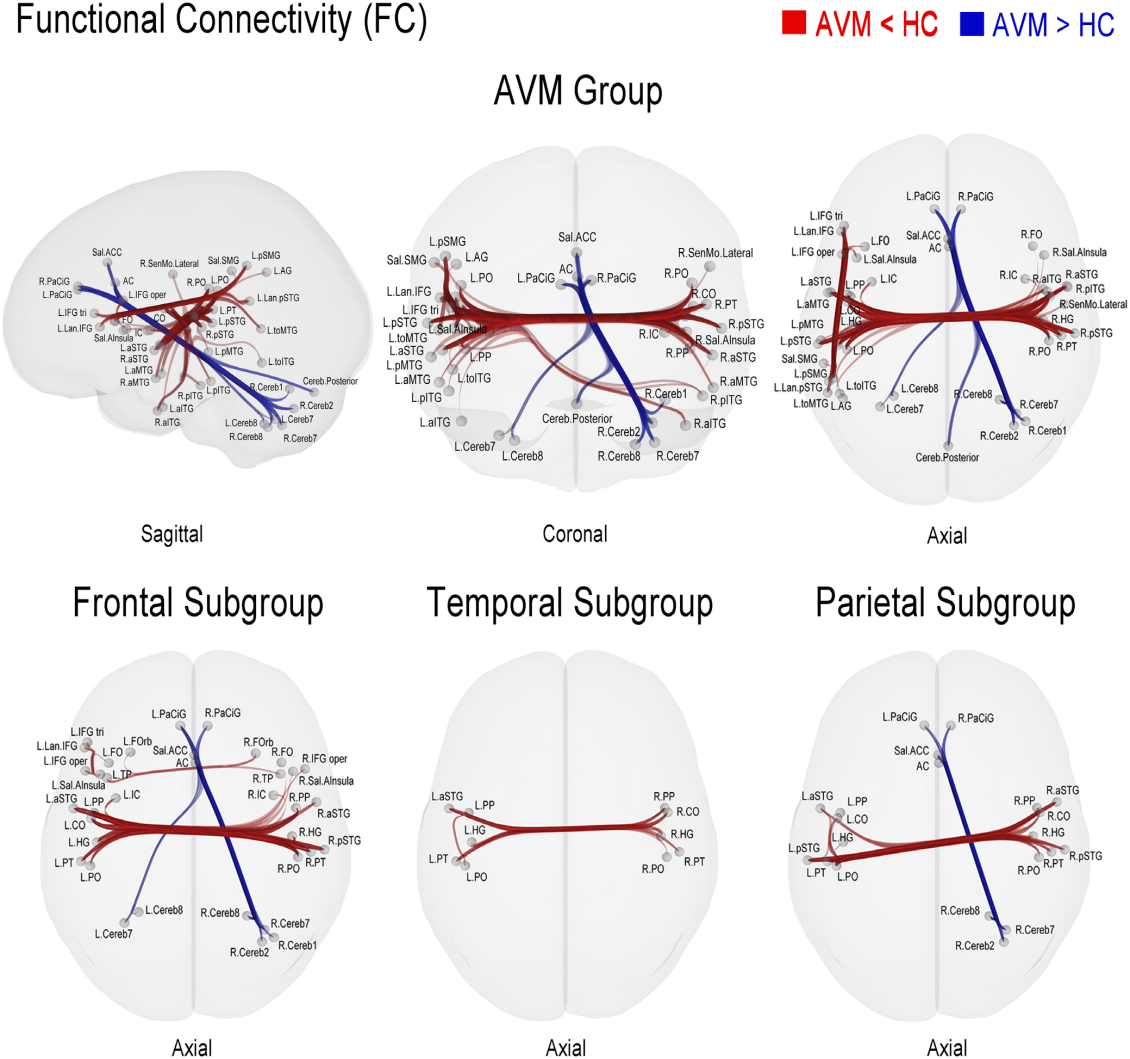
Results of FC analysis. Functional connectivity (FC) comparisons between healthy controls and AVM patients. Red lines indicate connections with significantly reduced FC in AVM patients versus controls; blue lines denote connections with significantly enhanced FC. The top row displays three projections of the AVM group brain map, while the bottom row shows, from left to right, the frontal, temporal, and parietal subgroup maps.

Overall, compared with the control group, the most obvious alteration of functional connectivity in the AVM group was the significantly decreased FC intensity between the language‐related brain regions in the left cerebral hemisphere and the corresponding brain regions in the right hemisphere (Figure 3). These decreased connections specifically included the FC between the left inferior temporal gyrus (ITG), middle temporal gyrus (MTG), angular gyrus (AG) and the right MTG and ITG, the FC between the bilateral superior temporal gyrus (STG), central opercular cortex (CO), parietal operculum cortex (PO), planum temporale (PT), Heschl's Gyrus (HG), planum polare (PP), and the FC between the left insular cortex and the right insular cortex, frontal operculum cortex (FO) and the inferior frontal gyrus (IFG).

Moreover, decreased FC intensity was also observed between the left SMG, STG, and the left IFG. Meanwhile, increased FC intensity was detected between the bilateral paracingulate gyrus (PaCiG), anterior cingulate (AC) cortex, salience, anterior cingulate cortex (Salience. ACC) and the bilateral cerebellar brain areas, including Cereb 8 (lobule VIII), 7 (lobule VIIb), 1 (Crus I) and 2 (Crus II).

#### 3.2.2 Results of the AVM Subgroups

The FC results of the frontal subgroup were similar to those of the AVM group (Figure 3). The main difference is that the descending FC in the left hemisphere mainly lies in the connections within IFG and between the insula and IFG, but not between the IFG and SMG or STG.

In the temporal subgroup, decreased FC intensity was observed between the left STG, PP, HG, PT and the right PP, HG, PT, PO, and CO. Descending FC intensity in the left hemisphere was observed between PO, PT, and STG. No significant increase in FC intensity was detected.

In the parietal subgroup, decreased FC intensity was observed between the bilateral STG, PP, HG, PT, PO, and CO. Descending FC intensity in the left hemisphere was observed between PO, PT, CO, and STG. Increased FC intensity was detected between the bilateral Pa CiG, AC, ACC, and the right Cereb 8, 7, and 2.

### 3.3 Results of TW‐sFC

#### 3.3.1 Results of the AVM Group

**FIGURE 4:**
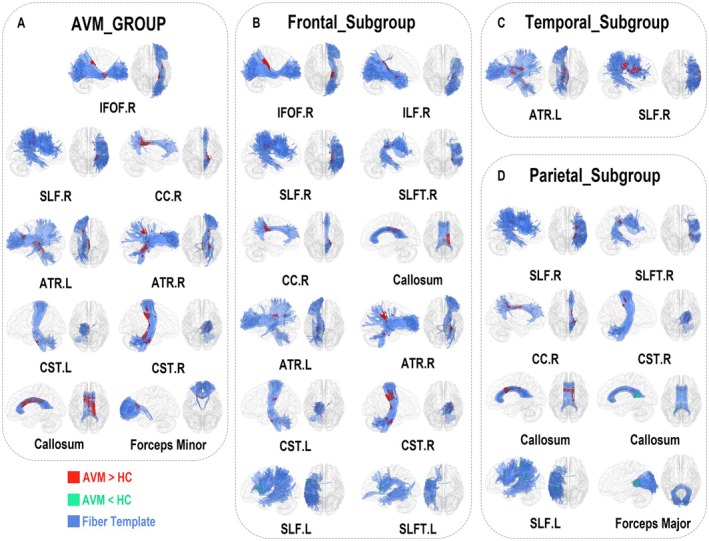
Results of TW‐sFC. Spatial comparisons of TW‐sFC intensity between healthy controls and AVM groups. (A) AVM group, (B) Frontal subgroup, (C) Temporal subgroup, and (D) Parietal subgroup. Within each frame, the subgraph displayed the TW‐sFC difference overlapped with the fiber template in sagittal and axial views. The blue regions indicate the fiber template ROI, while the red regions depict the areas that are significantly higher intensities in the AVM groups compared to healthy controls. Conversely, green regions denote areas with significantly lower intensities in the AVM group (only the number of voxels of TW‐sFC overlapped with fiber template over 200 were shown).

No significant decrease of TW‐sFC intensity was identified in the AVM group compared to the control group (Figure 4). In AVM patients, significant increases of TW‐sFC intensity were observed in the right IFOF, SLF, CC, ATR, and CST, and also in the left ATR and CST. Moreover, the callosum (body section) and the forceps‐minor also showed an increase in TW‐sFC intensity.

#### 3.3.2 Results of the AVM Subgroups

In the frontal subgroup, a significant decrease of TW‐sFC intensity was observed in the left SLF and SLFT. There was a significantly increased TW‐sFC intensity in the right IFOF, ILF, SLF, SLFT, ATR, CC, CST, the left ATR and CST, and the callosum.

In the temporal subgroup, no significant decrease was observed. Increased TW‐sFC intensity was detected in the left ATR and right SLF.

In the parietal subgroup, there was a significantly decreased TW‐sFC intensity in the splenium section of the callosum, the left SLF, and the forceps major. Meanwhile, increased TW‐sFC intensity was found in the right SLF, SLFT, CC, CST, and the callosum (body section).

## 4 Discussion

### 4.1 Language Remodeling Patterns Revealed by FC Analysis

The results of FC analysis can be decomposed into three parts. The first part showed a decrease of FC intensity between different brain regions in the left hemisphere. This is trivial to understand because the lesion impairs the language areas in the left hemisphere, leading to a decrease of FC intensity between different language areas in the left hemisphere. The second part was a decrease of FC intensity between bilateral cerebral hemispheres, especially between the language areas in the left hemisphere and their corresponding homotopic areas in the right hemisphere. The third part showed an enhancement of FC intensity between the anterior cerebral midline brain areas and language‐related brain regions in both cerebellar hemispheres. We propose that the results of the latter two parts may be related to language function remodeling in AVM patients.

#### 4.1.1 Decrease of FC Intensity Between the Bilateral Cerebral Hemispheres

We believe that the decrease in FC intensity between the bilateral hemispheres may be related to the weakened inhibitory effect of the left dominant hemisphere on the right hemisphere. It is speculated that an inhibitory relationship exists between homologous brain regions across the bilateral hemispheres [33, 34, 35]. In most healthy brains, inhibition from the left to the right is predominant to maintain language dominance in the left cerebral hemisphere [36]. Meanwhile, the right hemisphere also has an inhibitory effect on the left hemisphere [37]. In AVM patients, when the dominant left language areas are impaired by lesions, the inhibitory effect from the left hemisphere language areas on the corresponding right‐hemisphere areas is weakened [13, 38, 39]. As a result, homotopic brain areas in the right hemisphere may activate their suppressed and potential language functions, partially compensating for lost functions in the left hemisphere. This is consistent with the results of our previous study regarding the ALFF alterations of rs‐fMRI in AVM patients, in which we found ALFF values significantly increased in the anterior part of the right putamen in the frontal subgroup, in the posterior part of the right inferior and middle temporal gyrus in the temporal subgroup, and in the right inferior parietal lobule in the parietal subgroup [12]. Therefore, we believe the decrease in the inter‐cerebral functional connectivity reflected the weakened inhibitory effect from the language areas of the left hemisphere to the corresponding brain areas of the right side.

Interestingly, although different AVM subgroups showed distinct functional connectivity alteration patterns, we found one common pathway of FC intensity decline, which lies between the bilateral STG, PP, HG, PT, PO, and CO. These brain areas were all located around the posterior part of the lateral fissure and were supposed to be associated with language functions [40, 41, 42].

#### 4.1.2 Increase of FC Intensity Between the ACC and Cerebellar Hemispheres

It is known that the cerebellum plays an important role in language. Although its exact function remains controversial, the cerebellum is believed to be engaged in regulating the functions of the cerebral language regions [43]. Meanwhile, in a healthy brain, the right cerebellar hemisphere is dominant for language, with the lateral part of the right posterior cerebellar lobe—including lobule VI, Crus I, Crus II, lobule VIIB, and lobule VIII—serving as the primary language area [44]. In our previous research on rs‐fMRI in AVM patients, we found that the cerebellum played an important role in language compensation, especially the left lobule VIII [12]. However, the mechanism by which these cerebellar areas interact with the cerebral cortex in AVM patients remains unclear. According to the results of the current study, we found the bilateral Crus I, Crus II, lobule VII, and lobule VIII had a significantly increased FC with the anterior cingulate gyrus and its surrounding brain areas, including the bilateral Pa CiG, AC, and Salience ACC. Similar findings were found in the frontal and parietal subgroups, but not in the temporal subgroup, which might be caused by a limited sample size.

In a bilingual language control study, Yuan et al. found that the cerebrocerebellar connection exists between dorsal ACC (dACC) and cerebellar lobule VI. Additionally, they revealed that the dACC is connected to the cerebral language cortex, whereas lobule VI is connected to lobule VIII [45]. The above results indicate that in healthy individuals, the cerebellar language‐related areas interconnect with and regulate the cerebral language regions through ACC. In AVM patients, we speculate that this functional connectivity is enhanced. The cerebellum upregulates the language functions of the cerebral hemispheres through ACC, thereby maintaining the intact language function of AVM patients.

### 4.2 Language Remodeling Patterns Revealed by TW‐sFC

Within the AVM group, significant increases of TW‐sFC intensity were found in fiber bundles (IFOF, SLF, and ATR) of the right cerebral hemisphere and the left ATR. No significant decrease was observed. Meanwhile, the callosum also presented with TW‐sFC intensity increase, which might reflect enhanced intracerebral connection. Moreover, different AVM subgroups showed different change patterns of the TW‐sFC intensity. The TW‐sFC intensity decreased in the left fiber bundles in the frontal and parietal subgroups, which was assumed to be attributed to the impairment caused by the AVM lesions.

We speculate that the TW‐sFC intensity increase in bilateral hemisphere fiber bundles has a protective effect on language function. Among them, the increase of TW‐sFC intensity in the left ATR suggested language remodeling of brain areas around the lesion in the left hemisphere. The TW‐sFC intensity increase in right fiber bundles may indicate language reorganization in the right hemisphere. This aligns with the findings from our previous studies regarding language reorganization in AVM patients. An ALFF study found an increase in ALFF values in the corresponding brain areas of the right hemisphere at resting state [12], and another research study on language‐related white matter plasticity using the AFQ method identified fiber bundle remodeling in both hemispheres [15].

### 4.3 Language Remodeling Mechanism of AVM Patients

**FIGURE 5:**
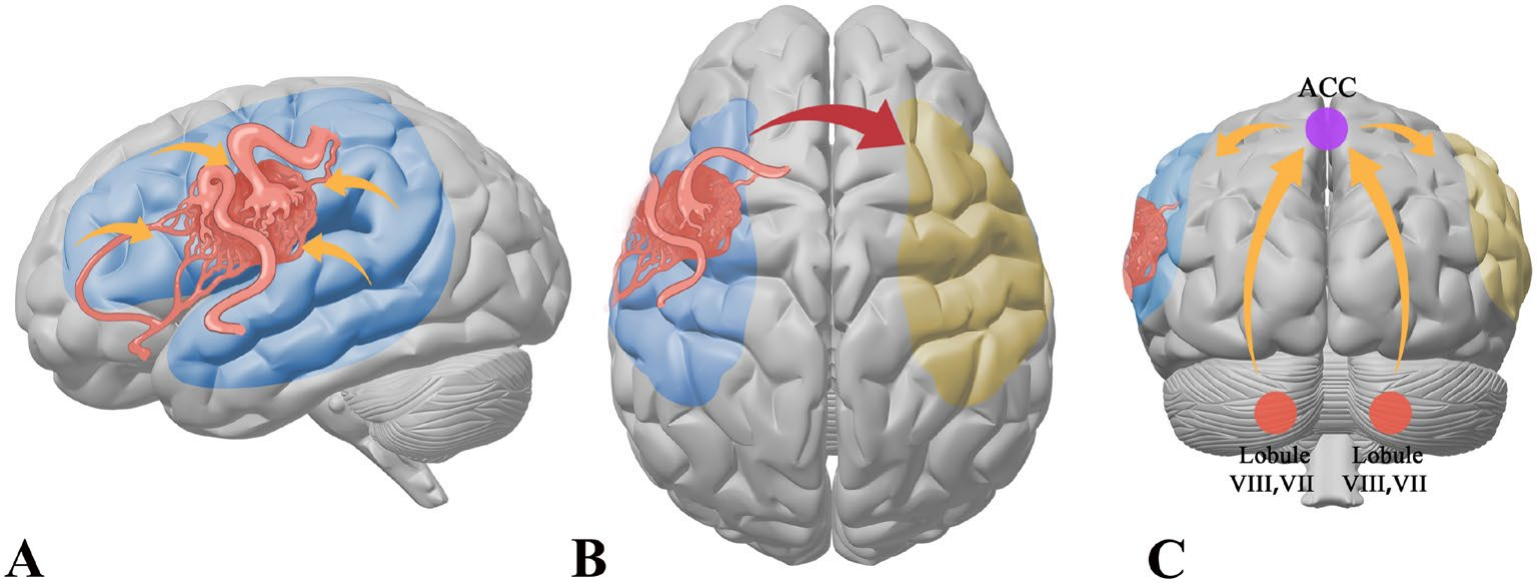
Language reorganization pattens of AVM patients. Three language reorganization patterns were shown in this figure, including reorganization of the brain areas surrounding the lesion in the left cerebral hemisphere (intracerebral connection reorganization) (A), the weakened inhibitory effect of the left cerebral hemisphere on the right cerebral hemisphere leading to activation of the potential language functions of the right cerebral hemisphere (inter‐cerebral connection reorganization) (B), and the upregulation of functions of the cerebral language areas by cerebellar language‐related brain regions through ACC (cerebrocerebellar connection reorganization).

In summary, three factors were found to contribute to the process of language function remodeling in AVM patients, including the brain cortex and fiber bundles surrounding the lesion in the left cerebral hemisphere (intracerebral connection reorganization), the homologous brain areas and fiber bundles of the traditional language areas in the right hemisphere (inter‐cerebral connection reorganization), and the cerebellum (cerebrocerebellar connection reorganization) (Figure 5).

Regarding the intracerebral connection reorganization, the remodeling of brain areas around the lesion in the left hemisphere is consistent with the results of our previous studies of the task‐based fMRI and DTI [13, 15], and will not be discussed in detail here. Regarding the interhemispheric connection reorganization, we speculate on the role of the right cerebral hemisphere in language remodeling in AVM patients based on the following evidence: the decreased functional connectivity between the left cerebral language areas and their right homologs; the increased TW‐sFC intensity of the fiber bundles in the right hemisphere; the increased ALFF values in the right hemisphere in our previous study [12]. As mentioned above, we speculate that the possible mechanism is that the inhibitory effect from the language areas of the left hemisphere to the corresponding brain areas of the right hemisphere is weakened, caused by the AVM lesion, and then the potential language function of the right cerebral homologous brain regions is activated. Moreover, the increased TW‐sFC intensity of the callosum also suggested enhanced inter‐cerebral connection, which might be the result of more involvement in the language function of the right hemisphere. Regarding the mechanism of the cerebrocerebellar connection reorganization, it is hypothesized that the cerebellum upregulates the language functions of both cerebral hemispheres through the ACC when the traditional language areas of the left cerebral hemisphere are impaired by the AVM lesions.

### 4.4 Limitations of the Study

The current study has several limitations. First, the small sample size may have an influence on the results. For example, no significant cerebrocerebellar connection alteration was found in the temporal subgroup, and the limited case number might be the reason. Second, as the language functions of all patients were normal, we were unable to investigate correlations between neuroimaging results and language test findings. In other words, although all the lesions we included involve the traditional language cortex, the findings of neuroimaging may not only be the result of language function remodeling, but may also include other cognitive function remodeling. Moreover, only the MRI data is analyzed in this study, and other methods could be applied to confirm the MRI findings; for example, transcranial magnetic stimulation (TMS) might be used to validate the language remodeling pathways that we have discovered in this study. Moreover, to keep consistency with the conventional static functional connectivity we used in this study, we applied the TW‐sFC to integrate the tractography and functional information. But there is also a dynamic version of this track weighted imaging method, track weighted dynamic functional connectivity (TW‐dFC). The alterations of TW‐dFC results between AVM patients and healthy controls might supply more information about AVM language remodeling.

## 5 Conclusions

By performing the FC and TW‐sFC analyses on both fMRI and dMRI datasets, we found three possible mechanisms of language reorganization in AVM patients. The first one is the reorganization of gray and white matter surrounding the lesion in the left cerebral hemisphere (intracerebral connection reorganization). The second one is the homologous gray and white matter in the right cerebral hemisphere, which is activated by the weakened inhibitory effect of the left cerebral hemisphere on the right cerebral hemisphere (inter‐cerebral connection reorganization). The third one is the reorganization of bilateral cerebellar language‐related brain regions, which works by upregulating the language functions of the cerebral hemispheres through ACC (cerebrocerebellar connection reorganization).

## Ethics Statement

This study was approved by the Institutional Review Board of Beijing Tiantan Hospital, Capital Medical University (number: KY2018–103–01).

## Consent

Written informed consent was obtained from all participants.

## Conflicts of Interest

The authors declare no conflicts of interest.

## Supporting information


**Table S1:** Intergroup differences in TW‐sFC between the AVM patients and healthy controls.

## Data Availability

The data that support the findings of this study are available from the corresponding author upon reasonable request.
