# Supplementary material for: Language Functional Connectivity Alterations During Resting State in Brain Arteriovenous Malformation Patients
Source: CNS Neurosci Ther. 2025 Sep 5;31(9):e70602. doi: 10.1111/cns.70602 (PMC12413485; doi:10.1111/cns.70602)
Supplement: Supplementary file 1 — Table S1: Intergroup differences in TW‐sFC between the AVM patients and healthy controls. [file CNS-31-e70602-s001.docx]

**Table S1:** Intergroup differences in TW-sFC between the AVM patients and healthy controls.

| **Brain Areas (AAL Label)** | **Distance** | **Voxels** | **MNI Coordinates** | | | **Peak Intensity** |
| --- | --- | --- | --- | --- | --- | --- |
|  |  |  | x | y | z |  |
| **AVM group** | | | | | | |
| Caudate_R | 1.41 | 12567 | 10 | 13 | 19 | 7.30 |
| SupraMarginal_R | 0 | 163 | 35 | -30 | 41 | 5.50 |
| Insula_R | 1 | 323 | 38 | -3 | -8 | 5.17 |
| Vermis_4_5 | 0 | 179 | -2 | -53 | -13 | 4.34 |
| Occipital_Mid_R | 1 | 196 | 50 | -80 | 9 | 4.32 |
| Cerebelum_3_R | 11.92 | 699 | 7 | -23 | -34 | 4.31 |
| Cerebelum_10_L | 16.55 | 178 | -4 | -20 | -38 | 4.27 |
| Supp_Motor_Area_R | 0 | 170 | 11 | -6 | 55 | 4.27 |
| Temporal_Pole_Mid_L | 0 | 210 | -24 | 9 | -37 | 4.26 |
| Postcentral_R | 0 | 238 | 29 | -33 | 64 | 4.10 |
| **Frontal Subgroup** |  |  |  |  |  |  |
| Cingulum_Post_L | 8.06 | 333 | -15 | -33 | 23 | 5.28 |
| Temporal_Sup_R | 0 | 219 | 52 | -7 | -13 | 5.05 |
| Cerebelum_3_R | 13.15 | 738 | 9 | -22 | -36 | 5.04 |
| Hippocampus_L | 0 | 155 | -25 | -12 | -11 | 4.67 |
| Cerebelum_3_R | 3.46 | 291 | 7 | -34 | -25 | 4.52 |
| Thalamus_L | 7 | 170 | -4 | -7- | -8 | 4.52 |
| Cingulum_Mid_R | 8 | 6390 | 6 | -10 | 29 | 4.43 |
| Cingulum_Mid_R | 0 | 317 | 13 | -7 | 47 | 3.94 |
| Occipital_Sup_L | 4.69 | 163 | -26 | -58 | 22 | 3.59 |
| Cerebelum_Crus2_R | 0 | 1216 | 18 | -86 | -42 | -6.20 |
| Cerebelum_Crus2_L | 0 | 676 | -12 | -88 | -34 | -5.58 |
| Cerebelum_Crus2_L | 0 | 246 | -34 | -84 | -33 | -5.57 |
| Frontal_Inf_Oper_L | 0 | 441 | -49 | 16 | 10 | -5.21 |
| Cerebelum_Crus1_R | 0 | 175 | 24 | -86 | -29 | -4.72 |
| Postcentral_L | 0 | 172 | -60 | 4 | 17 | -4.48 |
| Cerebelum_8_R | 0 | 211 | 29 | -71 | -48 | -4.24 |
| **Temporal Subgroup** |  |  |  |  |  |  |
| Insula_R | 1 | 285 | 37 | -10 | -6 | 7.49 |
| Caudate_L | 0 | 1871 | -8 | 10 | 16 | 6.59 |
| Caudate_R | 3.16 | 154 | 12 | -17 | 25 | 6.46 |
| Postcentral_R | 0 | 978 | 68 | -9 | 18 | 6.37 |
| Lingual_R | 2.44 | 153 | 22 | -104 | -4 | 6.14 |
| SupraMarginal_R | 1 | 473 | 69 | -31 | 31 | 6.14 |
| Insula_R | 0 | 151 | 34 | 31 | 0 | 5.92 |
| Cerebelum_6_R | 0 | 227 | 38 | -49 | -29 | 5.79 |
| Occipital_Mid_R | 0 | 209 | 49 | -81 | 8 | 4.74 |
| Thalamus_L | 7.28 | 430 | 0 | -26 | -5 | 4.57 |
| Cerebelum_9_L | 0 | 164 | -6 | -52 | -32 | 4.40 |
| Postcentral_R | 0 | 205 | 66 | 2 | 22 | 4.05 |
| Cerebelum_9_R | 12.08 | 157 | 2 | -51 | -70 | 3.87 |
| Amygdala_L | 0 | 220 | -23 | 1 | -18 | 3.69 |
| Thalamus_L | 0 | 560 | -9 | -15 | 2 | 3.25 |
| **Parietal Subgroup** |  |  |  |  |  |  |
| Vermis_4_5 | 0 | 448 | 0 | -47 | -15 | 5.64 |
| Caudate_R | 8 | 3545 | 1 | 5 | 18 | 4.95 |
| Cerebelum_4_5_R | 0 | 174 | 14 | -47 | -18 | 4.64 |
| Cerebelum_10_R | 11.36 | 157 | 11 | -24 | -44 | 4.41 |
| Frontal_Mid_R | 0 | 492 | 22 | 10 | 46 | 4.28 |
| Precentral_R | 2.24 | 339 | 30 | -10 | 47 | 3.94 |
| SupraMarginal_R | 2.45 | 184 | 42 | -37 | 33 | 3.75 |
| Postcentral_L | 0 | 173 | -35 | -37 | 60 | -5.32 |
| Temporal_Sup_L | 0 | 415 | -49 | -44 | 22 | -5.08 |
| Postcentral_L | 0 | 404 | -45 | -29 | 55 | -5.03 |
| Calcarine_L | 0 | 728 | -21 | -51 | 11 | -4.94 |
| Lingual_R | 0 | 222 | 19 | -96 | -8 | -4.82 |
| Rolandic_Oper_L | 0 | 192 | -41 | -16 | 22 | -4.61 |

Threshold: cluster-level p < 0.005, FWE-corrected. Distance refers to the spatial separation between the peak coordinate and the nearest brain region when the coordinate does not correspond to any region defined in the AAL atlas (e.g., white matter).
